# Enhancing antimicrobial surveillance in hospitals in England: a RAND-modified Delphi

**DOI:** 10.1093/jacamr/dlac092

**Published:** 2022-09-12

**Authors:** Selina Patel, Arnoupe Jhass, Susan Hopkins, Laura Shallcross

**Affiliations:** Institute of Health Informatics, University College London, London, England; Research Department of Primary Care & Population Health, University College London, London, England; UK Health Security Agency, London, England; Institute of Health Informatics, University College London, London, England

## Abstract

**Background:**

Optimizing antimicrobial use (AMU) is key to reducing antimicrobial-resistant infections, but current AMU monitoring in hospital provides limited insights for quality improvement.

**Objectives:**

To understand stakeholders’ priorities for developing national AMU surveillance in English hospitals to serve the needs of national policy makers and front-line practitioners.

**Methods:**

Characteristics of existing AMU surveillance systems were identified from a previous systematic review and categorized by the Acceptability, Practicability, Effectiveness, Affordability, Side-effects and Equity (APEASE) criteria. Stakeholders prioritized characteristics using a two-round RAND-modified Delphi (rating round 1, telephone panel discussion, rating round 2). Findings informed the design of a framework used to assess the extent to which existing surveillance approaches meet stakeholders’ needs.

**Results:**

Between 17/09/19 and 01/11/19, 24 stakeholders with national and local roles related to AMU prioritized 23 characteristics of AMU surveillance describing: resource for surveillance, data collection, data availability and pathways to translate information from surveillance into practice. No existing surveillance approaches demonstrated all prioritized characteristics. The most common limitation was failure to facilitate clinician engagement with AMU through delays in data access and/or limited availability of disaggregated metrics of prescribing.

**Conclusions:**

Current surveillance delivers national public health priorities but improving stewardship demands patient-level data linked to clinical outcomes. This study offers a framework to develop current surveillance to meet the needs of local stakeholders in England. Increased investment in data infrastructure and training is essential to make information held within electronic systems available to front-line clinicians to facilitate quality improvement.

## Introduction

In Europe, over 874 000 disability-adjusted life-years and 33 000 deaths have been attributed to antimicrobial resistant (AMR) infections in one year.^1^ Prior to the COVID-19 pandemic in England, one-fifth of bloodstream infections were drug resistant.^2^ Reducing antimicrobial use (AMU) in humans mitigates against the risk of AMR but, despite declining prescriptions in primary care, there has been a 7.8% increase in antibiotic use among hospital inpatients over 5 years. In 2020, this increasing trend reversed but this has been attributed to major changes in healthcare-seeking behaviour and mobility during the COVID-19 pandemic.^3^ Around 30% of prescriptions in secondary care may represent sub-optimal AMU.^4,5^ As well as reducing the risk of AMR, optimizing this use may improve patient outcomes and lead to financial savings in patient-care.^6,7^

Currently, AMU among hospital inpatients in England is estimated quarterly or annually, largely using aggregate metrics that do not capture the complexities of prescriber decision making such as patient case mix, disease severity and diagnostic uncertainty. The English Surveillance Programme of Antimicrobial Utilisation and Resistance (ESPAUR), which was established in 2014, reports annual estimates of total AMU (dispensed defined daily doses) per 1000 hospital admissions by antibiotic class and other antimicrobial groups of interest, based on aggregated data.^3^ Similarly, the Commissioning for Quality and Innovation (CQUIN) framework provides pay-for-performance incentives to improve antimicrobial stewardship (AMS) behaviours to optimize AMU, focusing on specific targets such as prescribing for lower urinary tract infection.^8^ These data are made publicly available and are well suited for national surveillance, but they provide little insight into the drivers of sub-optimal antibiotic prescribing at hospital level, which requires patient-level data linking prescriptions to diagnoses and clinical outcomes.^9^ It is challenging to identify how prescribing can be optimized in a specific hospital without data on groups of patients receiving antibiotics, duration of therapy, whether prescribing is congruent with guidelines, and how this varies across teams and specialties. Increasingly, this information is held by hospitals within electronic health records (EHRs), but it is rarely accessible to clinicians. Enhancement of national AMU surveillance to address these issues and meet the needs of both stakeholder groups has not yet been demonstrated.

Achieving this goal requires an understanding of the needs of different stakeholder groups involved in AMU surveillance at national, regional and local level, because surveillance approaches that are suitable to meet these needs will vary by context. The aim of this study was to identify expert stakeholders’ priorities regarding opportunities to enhance AMU surveillance in hospitals in England to meet the needs of both national policy makers and local hospital stakeholders. The extent to which existing surveillance addresses these needs was also evaluated, and potential approaches to meet unaddressed needs were identified. This was achieved using a RAND-modified Delphi process, which is an established mechanism for capturing views and identifying consensus among stakeholders.^10,11^

## Methods

**Figure 1.**
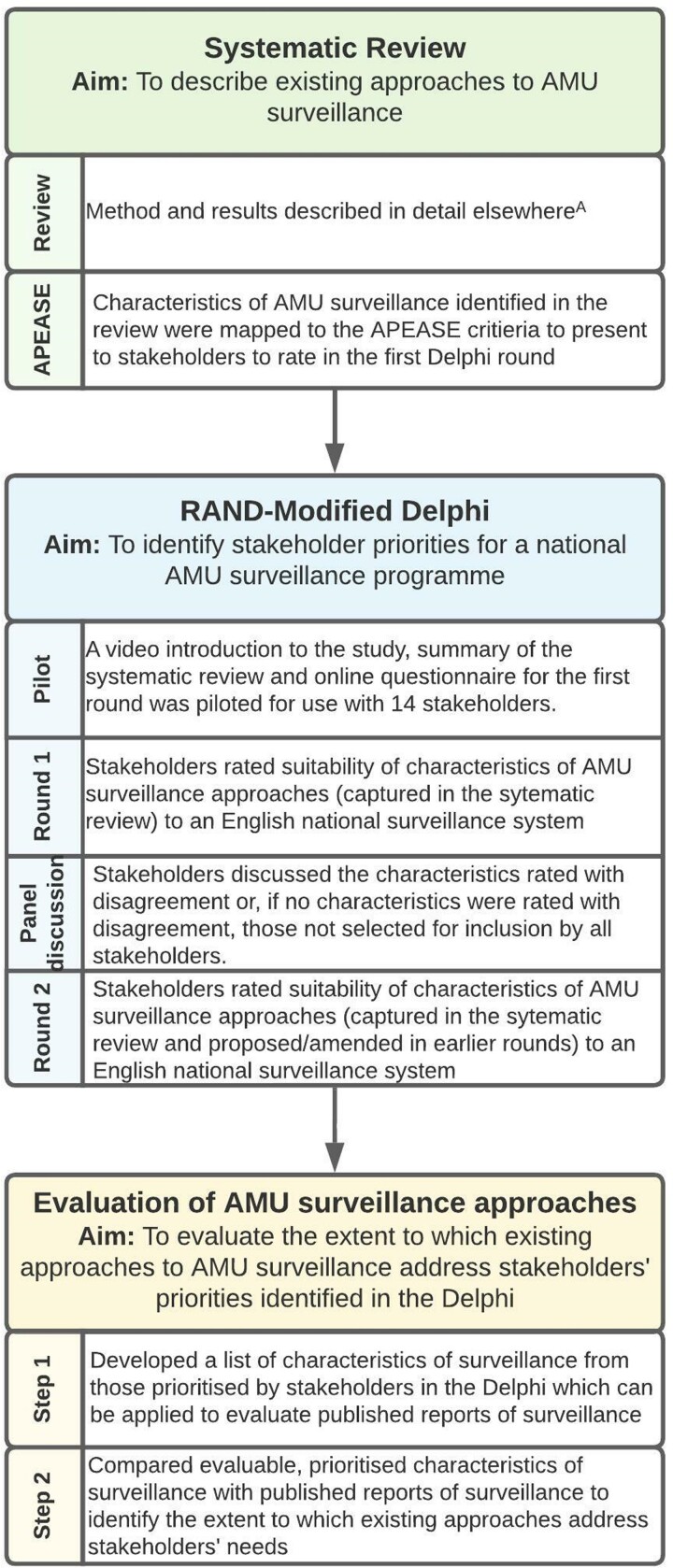
Description of the study methods.^16,40^^A^The method and results of the systematic review have been reported separately.^15^

This study was carried out in three consecutive stages (Figure 1):

- A systematic review of the literature to identify characteristics of existing surveillance systems, followed by an exercise to map these to the Affordability, Practicability, Effectiveness, Acceptability, Side-effects/safety and Equity (APEASE) criteria to facilitate consideration of the context-specific suitability of approaches.^12,13^
- A RAND-modified Delphi with local and national stakeholders to prioritize the mapped characteristics identified in (a) for inclusion in an enhanced national AMU surveillance strategy in England.
- Development of a framework based on the results of the Delphi (b) evaluate past and current surveillance approaches.

The following adheres to CREDES Delphi reporting guidelines.^14^

### Systematic review

The systematic review evidenced ways that AMU surveillance has been implemented to date and is reported elsewhere.^15^ Different characteristics of these, such as funding approaches or mechanisms to make the data available, were then mapped to the APEASE criteria (by S.P.). This mapping underwent two rounds of discussion and refinement between authors (S.P., L.S. and A.J.) to produce a list of characteristics of AMU surveillance that have been implemented.

### RAND-modified Delphi process

A two round online RAND-modified Delphi process with a telephone panel discussion was used to identify stakeholder priorities by rating the suitability of different approaches to implementing a national AMU surveillance system in English hospitals. Expert stakeholders involved in AMU surveillance locally and nationally were identified through the research teams’ existing networks spanning universities, hospitals, national organizations and government advisory groups. We aimed to achieve representation from all English NHS regions, include individuals from different types of hospitals (specialist and general) and capture the range of professions involved in stewardship (infectious disease physicians, microbiologists, pharmacists, public health), whilst keeping the number of participants suitable for a panel discussion.^16^ This study was approved by the University College London Research Ethics Committee (16765/001). Written informed consent was obtained from all participants and study materials were piloted before use (Figure 1). At each stage, stakeholders were given the opportunity to propose rephrasing or new characteristics for inclusion in the process.

#### Round 1

Stakeholders were sent an e-mail invitation to the study. They were provided with a video introduction to the study, an evidence summary based on findings from the systematic review, and an online questionnaire that asked them to rate the suitability of different ways to implement monitoring of AMU in hospitals in England. The questionnaire asked participants to rate these characteristics using a 9-point Likert scale (1, not at all well-suited to 9, extremely well-suited). Participants were asked to read the evidence summary and watch the video introduction before completing the questionnaire (see Supplementary data files at *JAC-AMR* Online).

#### Telephone panel discussion

Stakeholders were provided with a reminder of their Round 1 scores ahead of the panel discussion (Supplementary data). If the characteristics were rated with disagreement (≥30% responses falling in the top and bottom third of the Likert scale) in the first round, they were flagged for discussion at the panel telephone conference. If none of the characteristics were rated with disagreement, participants in the telephone conference were instead given the opportunity to comment on the characteristics that had not been selected (rated 7–9) by all of the panel, and to comment on additional characteristics that had been proposed by panel members in Round 1.^16^ If there was no misunderstanding or decision to rephrase a characteristic during the panel discussion, then characteristics with a median rating of 7–9 in Round 1 were selected for inclusion in Round 2 (as defined *a priori*).

#### Round 2

In Round 2, stakeholders were asked again to rate on a 9-point Likert scale the suitability of characteristics, which were selected or proposed in Round 1 and the panel discussion, for inclusion in a national approach to AMU surveillance. Characteristics with a median rating of 7–9 without disagreement were selected for inclusion in the final output.^16^

### Development of a framework to evaluate existing surveillance approaches

Next, the characteristics prioritized in the Delphi were translated into a framework that was applied to evaluate national and regional approaches to surveillance that had been previously implemented in high income countries. To ensure agreement, published studies identified through the systematic review and additional reports of existing surveillance systems were evaluated by two reviewers (S.P. and A.J.) in duplicate in batches of 15 (∼10%) references until a Kappa statistic of at least 0.6 was reached for inter-rater reliability of framework scores. A single author (S.P.) evaluated the remaining references using the framework. When it was uncertain if the study met the criteria, it was assumed that they did, except in the case of the data being made available for re-use for which it was decided that it would be clearly reported if the data were available. Similarly, when reports partially met the criteria, they were deemed to satisfy the criteria, except in the case of implementing digital versus manual surveillance as the inclusion of manual data collection for part of surveillance by definition implies that electronic surveillance could not be implemented. Results were reported as the proportion (with 95% CI) of reports/publications that fully or partially met each characteristic.

## Results

### Systematic review

The search terms for the systematic review returned 2736 records after duplicate removal, 145 of these met the study inclusion criteria (Figure S1). The results of the review are reported in more detail elsewhere.^15^ In addition, 22 reports of national-level surveillance that described 23 different approaches were identified (which had been excluded from the systematic review because they did not report a denominator). These were reported from Europe (11), North America (7), Oceania (4) and Asia (1). Nineteen characteristics were identified from studies identified through the systematic review and the additional national surveillance reports. These offered options to address the affordability, practicability, effectiveness, affordability, side-effects (unintended consequences) and equity of surveillance across sites (Table S1).

### RAND-modified Delphi

#### Expert panel members

Twenty-six stakeholders were invited to participate in the modified Delphi process, of whom 24 (92%) responded to the first questionnaire (17/09/19–30/09/19) and 14 (58%) participated in the panel discussion (09/10/19). Of these 24 Round 1 stakeholders, 21 (88%) also took part in Round 2 of the Delphi process (21/10/19–01/11/19) The 24 participants were Pharmacists (14), Microbiologists (8), an Infectious Diseases Consultant and a Public Health Consultant (Table S2). The average time since qualification was 22 years. Fourteen stakeholders were members of Trust/Regional advisory groups and committees, and 9 were members of national advisory groups or committees. The stakeholders worked at university, specialty and district general hospitals, as well as non-hospital settings including NHS Improvement and Public Health England, both nationally and across 5 of 7 NHS England regions (Tables S3 and S4). Eighteen participants (75%) worked at hospitals that used electronic prescribing or EHRs in patient care.

#### Characteristics of AMU surveillance prioritized for a national strategy in England

**Figure 2.**
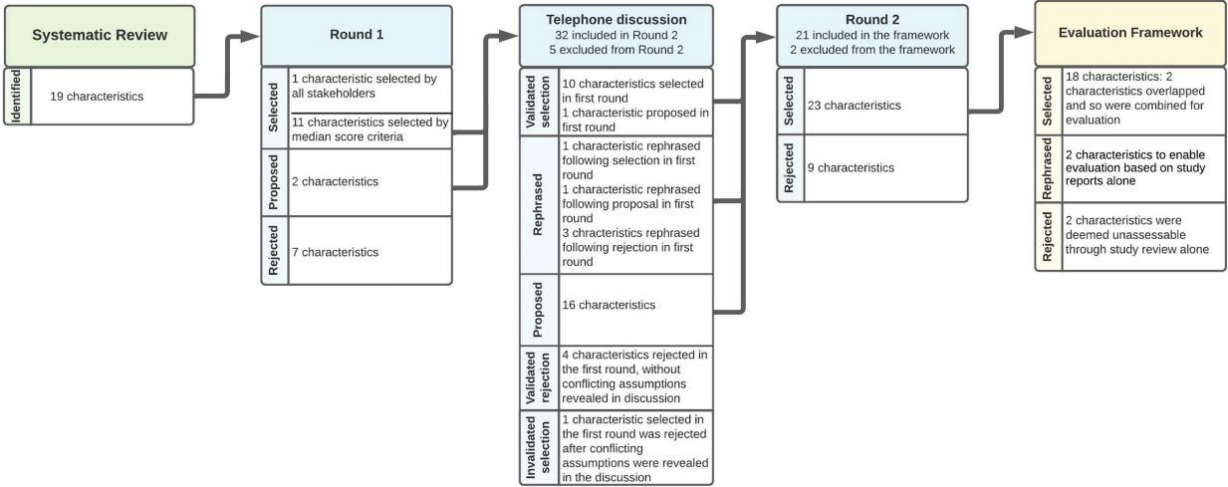
Flow chart of progression of characteristics of AMU surveillance during the Delphi. Characteristics were rated in Rounds 1 and 2 using a questionnaire with a 9-point Likert scale (1, not at all suited, to 9, extremely well-suited). No characteristics were rated with disagreement in Round 1, so stakeholders in the panel discussion were asked to comment on characteristics that were not rated 7–9 by all stakeholders and newly proposed characteristics. If there was no misunderstanding or decision to rephrase, then characteristics with a median score of 7–9 were included in Round 2. Characteristics prioritized by stakeholders were then translated into a framework to evaluate existing surveillance approaches to AMU surveillance.

In Round 1, 12/19 characteristics identified from the literature were selected and 2 more were proposed by stakeholders for inclusion in Round 2. None were rated with disagreement. In Round 2, stakeholders prioritized 23 characteristics; 10 were from Round 1, 10 were proposed in the telephone discussion and 3 were rephrased from earlier rounds (Figure 2). Selected characteristics spanned six themes – which describe the following (Table S5):

- Person-time and financial set-up resource – an investment (staff time and ring-fenced funding) to set up digital monitoring systems, rather than persisting with manual approaches.
- Data availability – the application of effective data governance and processes to make data available for monitoring and re-use within a week of data collection.
- Application of surveillance – the achievement of high coverage of surveillance despite differences in digital maturity between hospitals to draw comparisons of AMU over time and between settings.
- Flow of information – the development of relevant and representative metrics for reporting to different stakeholders.
- Translation of evidence into practice – the provision of pathways to improve patient care based on information obtained from AMU monitoring.
- Integration within the healthcare system – the contribution of surveillance to wider local and national quality improvement (QI) initiatives.

Fourteen characteristics were not prioritized and rejected by stakeholders in Rounds 1 and 2 (Table S6). These related to:

- Sustained person-time resource – a regular demand for staff time to carry out surveillance.
- Skills – a requirement for clinical training for data collection and local data science skills for analysis of use data.
- Untimely reporting – a 3-month lag from data collection to data availability.
- Ownership – an exclusively centralized approach to data collection and analysis, for example through a national agency.

### Evaluation of existing AMU surveillance against stakeholders’ priorities

Twenty characteristics from the Delphi process formed the basis for a framework, representing the six themes identified, to evaluate the extent to which published surveillance approaches meet the needs of local and national stakeholders (Figure 2 and Table 1).

**Table 1.** A framework to evaluate existing AMU surveillance based on study reports, derived from characteristics of national AMU surveillance prioritized by expert stakeholders

Theme	Evaluated characteristic (Y/N)	Explanation	Needs met
Resource	‘Ring-fenced’ funding provided.	Stakeholders identify funding and ‘making the business case’ as a barrier to surveillance.	Local
	Digital surveillance approach.	To obtain large and detailed enough datasets which can indicate patient case mix, disease severity and diagnostic uncertainty requires passive (recorded through patient care), electronic data collection.	Local/national/research
Data availability	Data including metrics available within a week of data collection.	To offer relevant information and effectively contribute to quality improvement, metrics must be available in a timely manner.	Local
	Data readily available for re-use.	To ensure surveillance datasets are available to meet national, local and research needs for surveillance.	Local/national/research
	Minimizes risk of a data breach.	To avoid losing the trust of the general public, which could halt surveillance, the risk of data breaches should be minimized through the implementation of good data governance, such as avoiding paper reporting systems.	General public
Application	Enables comparisons between specialties and hospitals.	To draw more accurate comparisons between settings and over time requires collecting variables to help adjust for differences between settings, such as patient case mix.	Local/national
	Monitors patient-level use over time.	To capture more accurate representations of AMU and inform targeted interventions to improve patient care requires detailed datasets over time.	Local/national
	Implementable across hospitals with varying levels of digital maturity.	To achieve greater coverage of surveillance requires an approach which is adaptable to different types of hospital.	National
Information	Clinician-level measures reported to the hospital.	To report relevant information to different stakeholders requires tailored reporting. For example, clinician-level metrics are useful for hospital quality improvement, but may have unintended consequences if included in national reporting. Additionally, local priorities for stewardship may sometimes vary from national trends.	Local
	Specialty-level measures reported to the hospital.		Local
	Specialty-level measures reported nationally.		National
	Hospital-level measures reported to the hospital.		Local
	Hospital-level measures reported nationally.		National
	Minimizes risk of misinterpreting the data e.g., consider information related to patient case-mix or don’t draw comparisons if this is unavailable.	Stakeholders identify a risk that hospitals with a greater need for AMU may be unfairly penalized by AMU improvement initiatives. To avoid this, and build stakeholder trust in metrics, the risk of misinterpreting the data should be minimized.	Local/national
Translate evidence into practice	Measures reported to high-level policy makers (who used them to inform prescribing decision-making).	To achieve impact from surveillance requires reporting to stakeholder networks which provide a pathway from surveillance information to impact.	National
	Measures reported to hospital-level stakeholders (who used them to inform prescribing decision-making).		Local
	Measures were reported to clinicians who used them to inform prescribing decision-making.		Local
	Evidence that implementing the system to monitor antimicrobial use in hospital leads to improved clinical outcomes.	For surveillance to improve patient outcomes, it should be implemented as part of antimicrobial stewardship and this should be monitored with outcomes.	Local/national
System not silo	The system to monitor antimicrobial use is integrated with ongoing improvement initiatives.	To maximize improvements in patient care, surveillance should be integrated as part of the local and national healthcare and public health system.	Local
	The system to monitor antimicrobial use supports national initiatives.		National

None of the existing surveillance approaches were compatible with all 20 of the characteristics in the framework (Supplementary data, APEASE results). The mean number of criteria met by studies captured in the systematic review was 10.5 (range: 5–16). For studies identified through additional reports of national surveillance, the median number of criteria met was 8 (range: 4–14).

**Figure 3.**
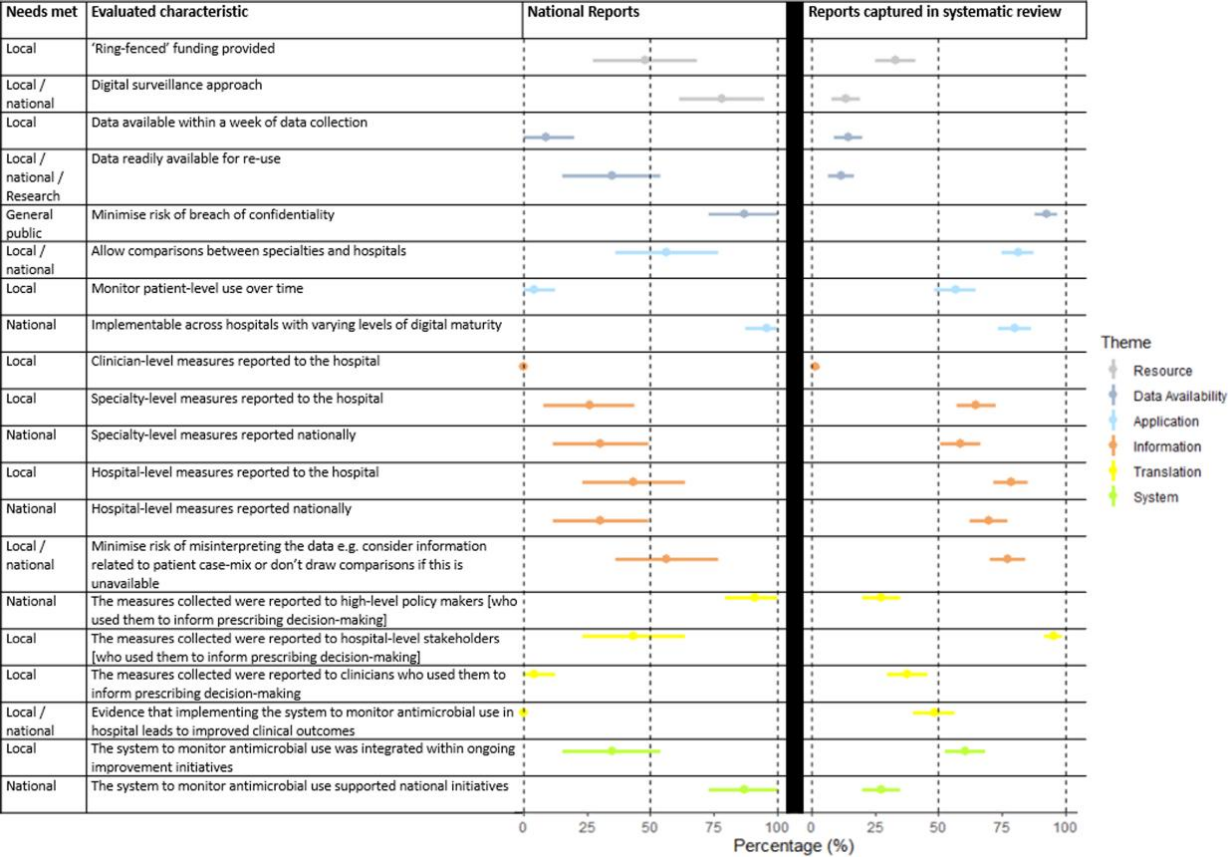
Proportion of surveillance approaches which addressed stakeholders’ priorities by type of needs met (those of local/national stakeholders) and AMU surveillance evaluation framework theme; person-time/financial set-up resource, data availability, application of surveillance, flow of information, translation of evidence into practice and integration within the healthcare system. 95% CIs are indicated. National reports less often addressed characteristics to meet the needs of local hospital teams and vice versa. National surveillance was often based on aggregated, digital datasets of AMU reported to national stakeholders. Reports captured in the systematic review more often reported manual approaches to obtain more detailed datasets and reporting of disaggregated metrics on prescribing e.g., patient-level or by specialty, which is needed to monitor local prescriber decision making.

Reports of national surveillance more often met national policy maker than hospital team needs (Figure 3). They were based on aggregated numerator data, such as dispensing obtained through electronic systems in place for patient care, and did not offer patient denominators. This approach facilitated estimates of total prescribing across a large number of hospitals that were often reported to national stakeholders monitoring use, but did not contribute to local quality improvement. Conversely, surveillance systems identified through the systematic review that included patient denominator data tended to report single site surveillance which more often met local than national stakeholder needs. These reports were based on patient-level data and disaggregated metrics of prescribing that could contribute to local quality improvement initiatives, but often were not reported to be fed back to national stakeholders or used to contribute to national surveillance initiatives. Furthermore, these more detailed data were more often obtained through manual review of patient notes and other sources, which does not lend itself to larger scale national surveillance.

### Enhancing surveillance in England

In England, the surveillance system described in the ESPAUR report represented most of the characteristics prioritized by stakeholders (14/20).^17^ Priorities that were not met included the collection of patient-level data (rather than aggregated), with timely availability (within a week) and the engagement of clinical stakeholders. No study or surveillance report evaluated using the derived framework (168/168) addressed all of these priorities simultaneously, but examples where some of them were met are described below.

#### Patient-level data

Approaches that digitally harnessed patient-level data (16/168) most frequently did so based on data already held within existing hospital systems such as hospital and clinical information systems or data warehouses (5/16),^7,18–21^ followed by electronic medical records (4/16),^22–25^ AMS databases developed in hospital (2/16),^26,27^ insurance databases (2/16)^28,29^ other routinely collected data (2/16)^30,31^ and for one approach this was unclear.^32^ Polk *et al.*^33^ was the only national-scale study without reported patient denominator that indicated surveillance based on patient-level data sources (hospital billing data in USA). However, the data extracted and analysed for surveillance purposes were aggregated to hospital-level.

#### Timeliness

Timely (within the week) feedback or data availability on prescribing was indicated in 23/168 studies. Four studies achieved this based on patient-level data with digital surveillance approaches. They either used in-house systems for antimicrobial stewardship with actionable feedback to physicians by pharmacists or physicians (3/4), or a real-time, regional (Ontario, Canada) database on critical care (Critical Care Information System) designed to improve responses to changes in patterns of service use including antimicrobial therapies (1/4).^26,7,20,25^ Studies such as Fukuda *et al.*^30^ digitally captured aggregate data on prescribing, but used physical ward rounds for patient-level information to provide timely, actionable feedback.

#### Engaging clinicians

Finally, the needs of clinicians were rarely met by global surveillance reports: 56/168 studies reported to clinical stakeholders in some way. Only 2/168 reported clinician-level measures to hospital stakeholders. Both characteristics were demonstrated in only one study, which was a quasi-experimental study of an audit and feedback intervention. This study was carried out in a single English University Hospital based on individual rates of antibiotic usage reported to doctors, as well as numbers of *Clostridioides difficile* and methicillin-resistant *Staphylococcus aureus* infection.^34^ The number of 7 day antibiotic courses per 100 admissions were reported monthly or less frequently (less often than prioritized by stakeholders) for 21 months and it was unclear who provided this feedback and how. Data collection was coordinated by junior doctors within each department.

## Discussion

Expert stakeholders prioritize AMU surveillance that addresses the needs of both national policy makers and local hospital teams, but few existing approaches achieve these two goals. In England, national AMU surveillance is mostly compatible with experts’ priorities but there is a major gap in access to timely, patient-level data that can be used to engage clinical teams in AMS. Our framework provides a mechanism to evaluate and refine existing surveillance approaches to ensure that they address the needs of all stakeholders who are engaged in AMS in hospital.

### Expert stakeholders’ priorities and existing national AMU surveillance

Stakeholders prioritize local and national AMU surveillance to facilitate continuous decision-making and quality improvement. Key domains that need to be considered are resourcing, data infrastructure/access/governance, the ability to produce metrics for different audiences that are comparable across settings and time, and the translation of this information into evaluable improvements in patient care. These priorities are in line with surveillance elements of AMS programmes prioritized in a previous Delphi carried out in 2018, and recommendations made in AMS guidelines from the CDC and NICE.^35–37^ There are existing studies, separate to this one, which consider which metrics should be applied in AMS initiatives.^10,11^ However, these need to be mapped to stakeholder groups and further developed and tailored to contexts to facilitate surveillance and ensure clinical utility as appropriate.

**Figure 4.**
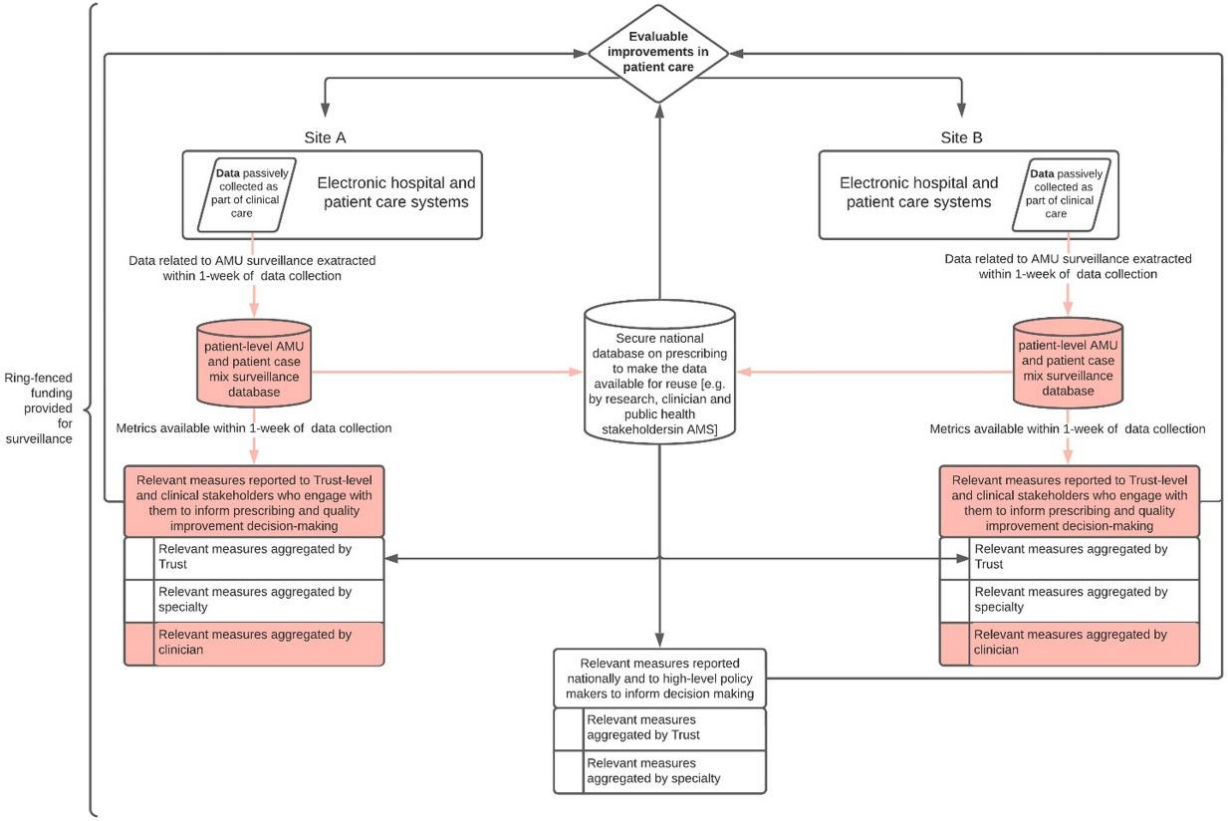
AMU surveillance in hospitals informed by expert stakeholders’ priorities. Key functions not addressed in existing national AMU surveillance programmes in England are highlighted in red. Prioritized characteristics describe passive data collection through digital systems for patient care. Data are extracted from these systems as local datasets on AMU for interrogation to address local priority areas for stewardship. Data from digital systems also contribute to integrated national surveillance datasets, which are made safely accessible for harnessing by researchers and other stakeholders in AMS. Relevant, standard metrics are extracted and are reported to clinical, other local and national stakeholders to contribute towards quality improvement in patient care. Against these key priorities, existing national surveillance in England is currently missing extraction of local datasets and feedback to engage local stakeholders.

Current national surveillance in English hospitals addresses many of these priorities but lacks patient-level data collection and reporting of disaggregated metrics to clinicians to engage front-line teams with subsequent quality improvement (Figure 4).^17^ A minority of evaluated surveillance approaches addressed these priorities, but those that did generally harnessed existing patient-level digital datasets from hospital patient-care systems, provided disaggregated data/information relevant to clinical practice, and offered personal delivery of individualized feedback to prescribers. In England, a minority of hospitals have digitized patient care. A subset of these have achieved this through electronic health records that offer detailed datasets for analysis that span admissions, investigations, procedures, microbiology, diagnoses and medications data.^38^ However, even with these datasets, interpreting antibiotic prescribing data can be challenging because of the structure of healthcare delivery. Care is usually provided by a team, which can make it difficult to attribute prescribing decisions to a specific individual (such as a junior doctor, consultant or microbiology, which offers specialist advice). Additionally, EHR data are often insufficient or structured in a way that makes retrospective assessments of concordance with prescribing guidelines complicated. Further work may be required to understand how to optimize the use of these datasets to support improvements in stewardship.

### Implications for policy

In England, patient-level data linked to clinical outcomes exist within hospital medical records (and increasingly EHRs) due to an existing policy commitment that aims to digitize patient care by 2024.^39^ However, these datasets are often not extracted as timely information for clinicians. Harnessing them could support the achievement of stakeholders’ priorities and requires investment in infrastructure and skills to establish pipelines to extract, integrate and analyse datasets from these systems locally and nationally. Metrics disseminated from these data should be defined through engagement with local hospital teams to identify those that offer insights relevant to both improving patient care locally (metrics for front-line practitioners, clinical directors, Trust Boards) and surveillance nationally. Demonstration of the importance of obtaining insights from health data during the COVID-19 pandemic coinciding with public health agency reform in England may provide an opportunity to formalize organizational and financial commitment to harness data from EHRs to simultaneously improve patient care locally and contribute to national surveillance.

### Limitations

Although good participation was achieved in the RAND-modified Delphi process from stakeholders involved in AMS in England, ideally a broader range of specialties would be involved in AMU surveillance and stewardship. Engagement from these specialties may result in different priorities for stewardship. A broader range of perspectives may have been achieved through a different sampling method, but this may have affected the high response rate. Secondly, identifying characteristics of surveillance related to the APEASE criteria and subsequent evaluations of surveillance approaches using the framework developed may have been limited by the information available in reports of surveillance alone. Additionally, the information available in reports varies with the purpose of surveillance. Group discussion and Kappa statistic assessment of evaluator agreement were used, respectively, to mitigate against the challenges of interpreting the information available. To avoid unfairly penalizing studies, the majority of uncertain results were assumed to meet the criteria. This may have led to an overestimate of the prevalence of some characteristics in surveillance approaches. Thirdly, stakeholders’ priorities in this study are composed of high-level descriptions of elements of surveillance and thus do not consider implementation. Rather, the derived framework for enhanced surveillance provides a tool to organize the integration of different AMS efforts and a common vision for stakeholders (including target-users as well as analysts and data architects) to work from in England.

### Conclusions

Expert stakeholders prioritize AMU surveillance systems that can address both local and national stakeholder needs for surveillance. Based on a framework for enhanced surveillance, current national AMU surveillance in England performs well against expert stakeholders’ priorities, but lacks local stakeholder engagement, which is required for AMU surveillance to contribute to local quality improvement. To facilitate this engagement, investment is required in software, training and infrastructure to make information from datasets that can support stewardship in clinical practice available to frontline practitioners.

## Supplementary Material

dlac092_Supplementary_Data

## Supplementary data

Tables S1 to S6, Figure S1 and further supporting documents are available as Supplementary data at *JAC-AMR* Online.
