# Supplementary material for: Enhancing antimicrobial surveillance in hospitals in England: a RAND-modified Delphi
Source: JAC Antimicrob Resist. 2022 Sep 12;4(5):dlac092. doi: 10.1093/jacamr/dlac092 (PMC9465639; doi:10.1093/jacamr/dlac092)
Supplement: dlac092_Supplementary_Data [file dlac092_supplementary_data.zip › 2022-075-Suppl-data.docx]

**
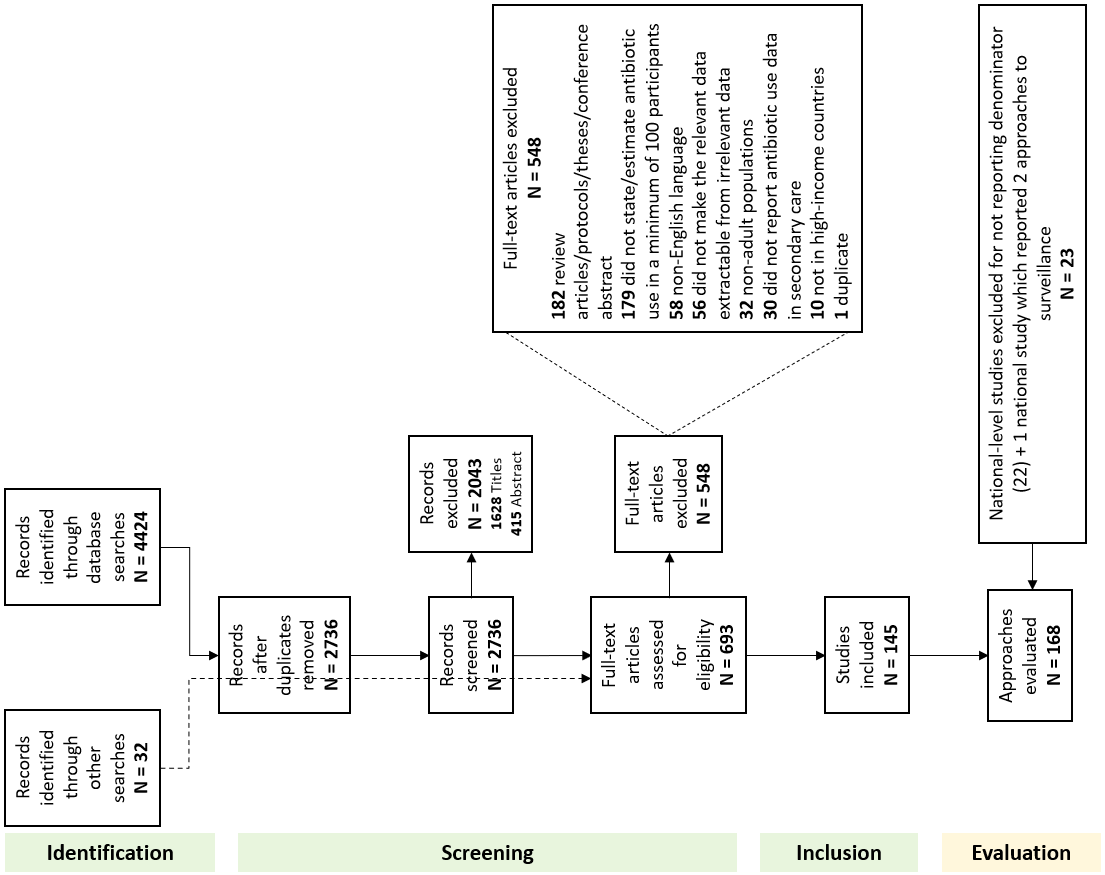
Supplementary data**

**Figure S1.** PRISMA Flow diagram of studies included in the systematic review and surveillance approaches which were evaluated against expert stakeholders' prioritised characteristics.

**Table S1.**Characteristics of surveillance extracted from published reports and studies of surveillance mapped to APEASE criteria.

| APEASE mapping | Characteristic identified from the literature |
| --- | --- |
| Practicability | Longer than a day required to establish the system in hospital to monitor antimicrobial use |
| Practicability | Longer than a day required to conduct the antimicrobial use surveillance once the monitoring system is in place |
| Affordability | High costs involved in the set up and maintenance of the system to monitor antimicrobial use in hospital |
| Practicability | A large number of people (>4) required to conduct surveillance in hospital |
| Practicability | Clinical training required to collect the data in hospital |
| Practicability | Local data analytical skills required to implement antimicrobial use monitoring in hospital, including professionals with the ability to use statistical packages such as R and STATA for analysis of big data sets on prescribing and patient care |
| Effectiveness | The system to monitor antimicrobial use could support existing initiatives such as CQUIN or national surveillance |
| Effectiveness | Evidence that implementing the system to monitor antimicrobial use in hospital leads to improved clinical outcomes |
| Effectiveness | The system to monitor antimicrobial use is integrated within existing hospital quality improvement initiatives such as reducing medication errors and improving sepsis outcomes |
| Effectiveness | The antimicrobial use surveillance data could be used to compare antimicrobial use across specialties and hospitals, for example by collecting variables to help adjust for differences between settings, such as patient case mix. |
| Effectiveness | The antimicrobial use surveillance system monitors patient-level use over time, meaning that it is possible to conduct longitudinal studies |
| Effectiveness | The measures collected are reported to high-level policy makers and they were used to inform decision making |
| Effectiveness | The measures collected are reported to Trust-level stakeholders who engaged with the surveillance system and used the measures to inform decision-making |
| Effectiveness | The measures collected are reported to clinicians who used them to inform prescribing decision-making |
| Side-effects | There is a lower risk of breach of confidentiality relating to patients, compared to other surveillance approaches, [through the implementation of good data governance such as avoiding paper reporting systems] |
| Acceptability | There is a lower risk of misinterpreting the data than other surveillance approaches [for example through the consideration of information related to patient case-mix or not drawing comparisons between settings if this is unavailable] |
| Side-effects | There is a lower risk of unintended consequences of surveillance on prescriber behaviour |
| Equity | The system to monitor antimicrobial use could be implemented across hospitals with varying levels of digital maturity |
| Affordability | It required the diversion of funds away from other areas of antimicrobial stewardship |

**Table S2.** RAND-modified Delphi response rate.

| Participants | Invited, n = 26 | Questionnaire 1, n = 24 | Group discussion, n = 14 | Questionnaire 2, n = 21 |
| --- | --- | --- | --- | --- |
| Pharmacist | 14 (54%) | 14 (58%) | 9 (64%) | 12 (57%) |
| Microbiologist | 8 (31%) | 8 (33%) | 3 (21%) | 7 (33%) |
| Infectious Diseases | 3 (12%) | 1 (4%) | 1 (7%) | 1 (5%) |
| Public Health | 1 (4%) | 1 (4%) | 1 (7%) | 1 (5%) |
| **Response rate %** | **-** | **92%** | **58%** | **88%** |

**Table S3.** Regions of the UK where participants in Round 1 of the RAND-modified Delphi work.

| **Professional region of the England** | **Number of participants, (n = 24)** |
| --- | --- |
| London | 9 (38%) |
| East of England | 1 (4%) |
| South East | 0 (0% |
| South West | 2 (8%) |
| Midlands | 3 (13%) |
| North East and Yorkshire | 7 (29%) |
| North West | 0 (0%) |
| National | 2 (8%) |

**Table S4.** Workplaces of participants in Round 1 of the RAND-modified Delphi.

| **Workplace** | **Number of participants, (n = 24)** |
| --- | --- |
| **University Hospital** | 14 (58%) |
| **Specialty Hospital (not university affiliated)** | 1 (4%) |
| **District General Hospital** | 7 (29%) |
| **Public Health England** | 1 (4%) |
| **NHS England and NHS Improvement** | 1 (4%) |

**Table S5**. Stakeholders' prioritisation of characteristics of surveillance approaches.

| **Characteristic** | | **Round 1** | | **Panel discussion** | **Round 2**  **Median score -> SELECTED/REJECTED** |
| --- | --- | --- | --- | --- | --- |
|  |  | **Median score** | **Expert selection -> progress** |  |  |
| 1 | Longer than a day was required to establish the system in hospital to monitor antimicrobial use | 5 | Selected by 7 experts -> validated in panel discussion | No misunderstanding or suggestion to rephrase  REJECTED | - |
| 2 | Longer than a day was required to conduct the antimicrobial use surveillance once the monitoring system is in place | 3 | Selected by 2 experts -> validated in panel discussion | No misunderstanding or suggestion to rephrase  REJECTED | - |
| 3 | High costs were involved in the set up and maintenance of the system to monitor antimicrobial use in hospital | 3 | Selected by 1 expert -> validated in panel discussion | No misunderstanding or suggestion to rephrase  REJECTED | - |
| 4 | A large number of people (>4) were required to conduct surveillance in hospital | 3 | Selected by 1 expert -> rephrased in panel discussion | REPHRASED: Professionals beyond the antimicrobial stewardship team at the Trust were required to conduct the surveillance | 5 -> REJECTED |
| 5 | Clinical training was required to collect the data in hospital | 5 | Selected by 3 experts -> rephrased in panel discussion | REPHRASED: Professionals with clinical expertise were required to collect the data in hospital | 6 -> REJECTED |
| 6 | Local data analytical skills were required to implement antimicrobial use monitoring in hospital, including professionals with the ability to use statistical packages such as R and STATA for analysis of big data sets on prescribing and patient care | 3 | Selected by 1 expert -> validated in panel discussion | No misunderstanding or suggestion to rephrase  REJECTED | - |
| 7 | The system to monitor antimicrobial use could support existing initiatives such as CQUIN or national surveillance | 9 | Selected by 22 experts -> validated in panel discussion | No misunderstanding or suggestion to rephrase | 8 -> **SELECTED** |
| 8 | There were an evidence base that implementing the system to monitor antimicrobial use in hospital leads to improved clinical outcomes | 9 | Selected by 23 experts -> validated in panel discussion | No misunderstanding or suggestion to rephrase | 8 -> **SELECTED** |
| 9 | The system to monitor antimicrobial use was integrated within existing hospital quality improvement initiatives such as reducing medication errors and improving sepsis outcomes | 9 | Selected by 20 stakeholders -> validated in panel discussion | No misunderstanding or suggestion to rephrase | 8 -> **SELECTED** |
| 10 | The antimicrobial use surveillance data could be used to compare antimicrobial use across specialties and hospitals | 8 | Selected by 20 stakeholders -> validated in panel discussion | No misunderstanding or suggestion to rephrase | 8 -> **SELECTED** |
| 11 | The antimicrobial use surveillance system monitored patient-level use over time, meaning that it is possible to conduct longitudinal studies | 8 | Selected by 20 stakeholders -> validated in panel discussion | No misunderstanding or suggestion to rephrase | 8 -> **SELECTED** |
| 12 | The measures collected were reported to high-level policy makers and they were used to inform decision making | 8 | Selected by 19 stakeholders -> rephrased in panel discussion | REPHRASED:  The measures collected were reported to high-level policy makers and they were used to inform decision making, whilst taking into account differences between sites such as patient case mix | 7 -> **SELECTED** |
| 13 | The measures collected were reported to Trust-level stakeholders who engaged with the surveillance system and used the measures to inform decision-making | 8 | Selected by 22 stakeholders -> validated in panel discussion | No misunderstanding or suggestion to rephrase | 8 -> **SELECTED** |
| 14 | The measures collected were reported to clinicians who used them to inform prescribing decision-making | 8 | Selected by 21 stakeholders -> validated in panel discussion | No misunderstanding or suggestion to rephrase | 8 -> **SELECTED** |
| 15 | There were a lower risk of breach of confidentiality relating to patients, compared to other surveillance approaches | 8 | Selected by 20 stakeholders -> validated in panel discussion | No misunderstanding or suggestion to rephrase | 8 -> **SELECTED** |
| 16 | There were a lower risk of misinterpreting the data than other surveillance approaches | 8.5 | Selected by 24 stakeholders -> validated in panel discussion | No misunderstanding or suggestion to rephrase | 8 -> **SELECTED** |
| 17 | There were a lower risk of unintended consequences of surveillance on prescriber behaviour | 8 | Selected by 18 stakeholders -> not validated in panel discussion | Due to misunderstanding. Characteristics 32 – 36 were proposed instead.  REJECTED | - |
| 18 | The system to monitor antimicrobial use could be implemented across hospitals with varying levels of digital maturity | 8.5 | Selected by 21 stakeholders | No misunderstanding or suggestion to rephrase | 8 -> **SELECTED** |
| 19 | It required the diversion of funds away from other areas of antimicrobial stewardship | 4.5 | Selected by 1 stakeholder -> rephrased in panel discussion | REPHRASED:  Funding which was ring-fenced for stewardship initiatives was provided to Trusts and this money was spent on developing antimicrobial use surveillance systems rather than other areas of stewardship | 7 -> **SELECTED** |
| 20 | The local antimicrobial stewardship team needed longer than a day to set up a manual survey in hospital to monitor antimicrobial use on an infrequent basis such as once or twice per year | - |  | PROPOSED | 5 -> REJECTED |
| 21 | Once set up, one person on each ward was required to manually survey quantitative measures of antimicrobial use | - |  | PROPOSED | 4 -> REJECTED |
| 22 | Once set up, the antimicrobial stewardship team manually surveyed the quality of antimicrobial use, for example through stewardship rounds as part of ongoing quality improvement at the Trust | - |  | PROPOSED | 5 -> REJECTED |
| 23 | Longer than a day and professionals across the hospital were required to set up a digital system to monitor antimicrobial use on a frequent basis which can be used regularly for quality improvement | - |  | PROPOSED | 7 -> **SELECTED** |
| 24 | One morning of work by one member of staff was required to implement weekly digital audits of antimicrobial use, after the system had already been set up | - |  | PROPOSED | 6.5 -> REJECTED |
| 25 | For manual data entry, some level of peer review was implemented to ensure data integrity | PROPOSED |  | No misunderstanding or suggestion to rephrase | 6 -> REJECTED |
| 26 | Data collected were analysed centrally, for example by a coordinating organisation such as Public Health England, rather than locally | - |  | PROPOSED | 5 -> REJECTED |
| 27 | The data were made available for quality improvement within 3 months of the data collected | - |  | PROPOSED | 5.5 -> REJECTED |
| 28 | The data were made available for quality improvement within the same week of the data being collected | - |  | PROPOSED | 8 -> **SELECTED** |
| 29 | The data were made available for quality improvement the same day as the data being collected | - |  | PROPOSED | 8 -> **SELECTED** |
| 30 | The data collected contributed to a secure, anonymised national database on prescribing which those involved in stewardship could use for quality improvement at a local and national level | - |  | PROPOSED | 8 -> **SELECTED** |
| 31 | The data collected could contribute to metrics collected at a national level (to support high level policy) as well as at a local level (to support local quality improvement) | - |  | PROPOSED | 8 -> **SELECTED** |
| 32 | It produced clinician-level measures for local (Trust) reporting and quality improvements | - |  | PROPOSED | 8 -> **SELECTED** |
| 33 | It produced specialty level measures for national reporting | - |  | PROPOSED | 8 -> **SELECTED** |
| 34 | It produced specialty level measures for local (Trust) reporting | - |  | PROPOSED | 8 -> **SELECTED** |
| 35 | It produced Trust level measures for national reporting | - |  | PROPOSED | 8 -> **SELECTED** |
| 36 | It produced Trust level measures for local (Trust) reporting | - |  | PROPOSED | 8 -> **SELECTED** |
| 37 | Demonstrated compatibility with all electronic prescribing systems currently available in the UK | PROPOSED |  | REPHRASED:  For those with digital systems for prescribing, it had demonstrated compatibility with all electronic prescribing systems currently available in England | 8 -> **SELECTED** |

As no characteristics were rated with the *a priori* definition of disagreement, characteristics which were not selected (rated in the top tertile) by all stakeholders were flagged for discussion in the telephone conference to validate responses and ensure there was no artefactual disagreement between stakeholders. In the absence of any misunderstandings or suggestions to rephrase, the characteristic was selected or rejected according to the criteria decided upon prior to starting the study. Round 1 n = 24. Telephone panel discussion n = 14. Round 2 n = 21

##

**Table S6.** Characteristics which were not prioritised and rejected by stakeholders in Rounds 1 and 2.

| **Theme** | **Evaluated characteristic** |
| --- | --- |
| Sustained person-time resource | Longer than a day required to establish the system in hospital to monitor antimicrobial use |
|  | Longer than a day required to conduct the antimicrobial use surveillance once the monitoring system is in place |
|  | A large number of people required to conduct surveillance in hospital |
|  | The local antimicrobial stewardship team need longer than a day to set up a manual survey in hospital to monitor antimicrobial use on an infrequent basis such as once or twice per year |
|  | Once set up, one person on each ward required to manually survey quantitative measures of antimicrobial use |
|  | Once set up, the antimicrobial stewardship team manually survey the quality of antimicrobial use, for example through stewardship rounds as part of ongoing quality improvement at the Trust |
|  | One morning of work by one member of staff required to implement weekly digital audits of antimicrobial use, after the system had already been set up |
|  | For manual data entry, some level of peer review implemented to ensure data integrity |
|  | High costs involved in the set up and maintenance of the system to monitor antimicrobial use in hospital |
| Skills | Clinical training required to collect the data in hospital |
|  | Local data analytical skills required to implement antimicrobial use monitoring in hospital, including professionals with the ability to use statistical packages such as R and STATA for analysis of big data sets on prescribing and patient care |
| Ownership | Data collected analysed centrally, for example by a coordinating organisation such as Public Health England, rather than locally |
| Untimely reporting | The data made available for quality improvement within 3 months of the data collected |
|  | A lower risk of unintended consequences of surveillance on prescriber behaviour |
