# Supplementary material for: Enhancing antimicrobial surveillance in hospitals in England: a RAND-modified Delphi
Source: JAC Antimicrob Resist. 2022 Sep 12;4(5):dlac092. doi: 10.1093/jacamr/dlac092 (PMC9465639; doi:10.1093/jacamr/dlac092)
Supplement: dlac092_Supplementary_Data [file dlac092_supplementary_data.zip › Suppl1_Round1_online_questionnaire (4).pdf]

Questionnaire 1: Antimicrobial Use Surveillance In Hospitals

Thank you for taking the time to complete this survey as part of a Delphi process on methods for antimicrobial use surveillance in hospitals in England.

Please click on the video link to watch the introduction to the survey (subtitles are provided)

In a systematic review of the literature, over 100 quality and quantity measures of antimicrobial use have been identified (different combinations of numerators and denominators). This variation contributes to the incomparability of estimates of antimicrobial use which are needed to benchmark progress towards controlling the risk of the emergence and spread of antibiotic resistance. This variation may be driven by a lack of consensus on antibiotic use metrics, as well as differences in the availability of data and approaches to surveillance across settings.

Please read the information in the key-findings pdf attached to the email invitation to complete this survey which summarises the key findings of this systematic review of the literature. Only after reading this pdf, please then complete the following survey which aims to examine the suitability of existing approaches to monitor adult, systemic antimicrobial use in hospitals in England for application in a nationally standardised approach to antimicrobial use surveillance.

For each question in the survey select how well-suited each characteristic of antimicrobial use monitoring approaches are for application in a national surveillance strategy. For example, this may consider factors such as differences in digital maturity and resource availability for data collection across sites, or the professionals required to conduct this surveillance work. Please respond using the information in the pdf and drawing on your knowledge and experience of working in NHS hospitals in England.

Please look out for invitations to participate in the group telephone discussion on 9th October and the follow up survey on 21st October!

If you have any questions regarding this survey please contact:

Selina Patel, PhD student

Institute of Health Informatics | UCL

Email: selina.patel.17@ucl.ac.uk

Telephone: 020 3549 5042

Consent

Consent form for professionals in the Delphi process to inform the development of a standardised approach to monitoring antimicrobial use surveillance in hospitals in England.

This study has been approved by UCL Research Ethics Committee, ID number: 16765/001

I confirm that I understand that by selecting each box below I am consenting or not consenting to this element of the study. I understand that not giving consent for any one element may mean that I am deemed ineligible for the study.

I confirm that I have read and understood the study information sheet (attached to the email invitation for this survey). I have had the opportunity to consider the information and what is expected of me, as well as ask any questions which have been answered to my satisfaction.<br>I would like to take part in the following (please tick if you would like to take part, regardless of availability):	☐ Questionnaire 1 (this questionnaire)<br>☐ Group telephone discussion<br>☐ Questionnaire 2<br>☐ none
I understand that I will be able to withdraw my relevant data up to 2 weeks after each stage of the Delphi process	◯ Yes<br>◯ No
The personal information you choose to provide: name, profession, job title, years since qualification, type of hospital (if applicable) and membership of local and national antimicrobial stewardship advisory groups, will be used to describe the participant group for this Delphi process for scientific publication. Additionally, your name will also be used to provide a personalised reminder of your responses to this survey compared to the median response of participants, ahead of the group discussion. This will be sent to you via direct email and will not be shared with any of the other participants.<br>Please type '.' if you do not wish to provide this information.<br><br>I consent to the processing of the personal information provided as described. I understand that such information will be handled in accordance with all applicable data protection legislation.	◯ Yes<br>◯ No
I consent to my name being published as a participant in the outputs of this research.<br><br>Please not that if you select yes to the processing of your personal information to describe the participant group in the question above, but do not consent to your name being given as a participant in the outputs of this research, your name will not be published but all other professional information published in the analysis to describe the participant group.	◯ Yes<br>◯ No
I understand that participation in this process will not be confidential and that the participant group will be described in the analysis. However, I understand that my responses to the surveys will be kept confidential.	◯ Yes<br>◯ No
The researchers will also maintain confidentiality for comments made during the group discussion and other participants will be asked to to the same. However, for this reason, I understand that confidentiality cannot be guaranteed for comments made during the group discussion.	◯ Yes<br>◯ No

I understand that participation is voluntary and I am free to withdraw at any time without giving a reason. I understand that if I decide to withdraw, any personal data I have provided up to that point will be deleted unless I agree otherwise.	◯ Yes<br>◯ No
I understand the potential risks of participating	◯ Yes<br>◯ No
I understand the direct/indirect benefits of participating	◯ Yes<br>◯ No
I understand that I will not benefit financially from this study or from any possible outcome it may result in the future	◯ Yes<br>◯ No
I understand that the information I submit will be published as a report	◯ Yes<br>◯ No
I consent to the group telephone discussion being recorded. I understand that recordings will be stored on the secure UCL data safe haven until the project is complete, when it will be deleted.<br><br>Please note: if you do not want your participation recorded you can still take part in the study	◯ Yes<br>◯ No
I am aware of who I should contact if I wish to lodge a complaint	◯ Yes<br>◯ No
I voluntarily agree to take part in this study	◯ Yes<br>◯ No
Please type your name to identify and confirm that this is your consent form	__________________________________

Respondent information

What kind of professional are you?	◯ Pharmacist<br>◯ Physician<br>◯ Surgeon<br>◯ Microbiologist (SpR or above)<br>◯ Nurse<br>◯ Infection control practitioner<br>◯ Other
Please specify	__________________________
What is your job title(s)?	________________________________
For how many years have you been qualified?	__________________________
Are you a member of any national or local advisory panels on antimicrobial stewardship?	◯ Yes<br>◯ No
Please list	________________________________
Which region of England do you work in?	◯ East of England<br>◯ London<br>◯ Midlands<br>◯ North East and Yorkshire<br>◯ North West<br>◯ South East<br>◯ South West<br>◯ National
Where do you work?	◯ District general hospital<br>◯ Specialty hospital (not university affiliated)<br>◯ University hospital<br>◯ Government agency<br>◯ other
Please specify	__________________________
Does your hospital use electronic prescribing or electronic health records for patient care?	◯ Yes<br>◯ No<br>◯ N/A
Your name	__________________________

Page 1/9

Information pack

Is there anything you did not understand in the pdf of information?

____

Any other feedback about the pdf of information

____

Page 2/9

Resources required for surveillance

Please consider the information about existing AMU surveillance strategies captured in the systematic review and your knowledge and experience of working with the NHS to answer these questions.

For example, when answering the questions you may want to consider your existing knowledge of differences in the level of resource available for AMU surveillance across hospitals in England, as well as the information captured in the systematic review about level of resource required for surveillance strategies. Resources required and resources available may be one of many factors which influence how suitable it would be to implement a coordinated surveillance strategy across hospitals with each of the following characteristics.

When designing a national antimicrobial use surveillance system for implementation across hospitals in England, how well-suited would the system be if:

---

1 Longer than a day was required to establish the system in hospital to monitor antimicrobial use

- ◯ 1 not at all suited
- ◯ 2
- ◯ 3
- ◯ 4
- ◯ 5 neither well-suited or not
- ◯ 6
- ◯ 7
- ◯ 8
- ◯ 9 extremely well-suited
- ◯ unsure

(For example, the findings from the systematic review were that one hospital required 16 hours to plan a point prevalence survey. Conversely, algorithms which estimate use and assess appropriateness are complex to develop and may need to be developed to be site specific using the data and guidelines available.)

---

2 Longer than a day was required to conduct the antimicrobial use surveillance once the monitoring system is in place

- ◯ 1 not at all suited
- ◯ 2
- ◯ 3
- ◯ 4
- ◯ 5 neither well-suited or not
- ◯ 6
- ◯ 7
- ◯ 8
- ◯ 9 extremely well-suited
- ◯ unsure

(For example, digital surveillance using electronic prescribing systems requires an initial time investment to set up, i.e. writing scripts to extract and analyse the data. However, once these scripts are written, it should not take more than a morning to run the analysis to conduct surveillance each time the Trust requires a report on antimicrobial use. Conversely, manual surveys captured in the systematic review estimated 5-20 minutes required to extract data per patient included for surveillance, with a further 16 hours for data cleaning and analysis.)

3	High costs were involved in the set up and maintenance of the system to monitor antimicrobial use in hospital	◯ 1 not at all suited<br>◯ 2<br>◯ 3<br>◯ 4<br>◯ 5 neither well-suited or not<br>◯ 6<br>◯ 7<br>◯ 8<br>◯ 9 extremely well-suited<br>◯ unsure<br>(For example, is it feasible for all hospitals to meet the additional costs required to set up and maintain e-prescribing and electronic health record systems for AMU surveillance, rather than using point prevalence surveys for less frequent surveillance. Furthermore, professionals trained in using statistical packages such as R or SPSS would be necessary to analyse these big data sets.)

Page 3/9

Implementing surveillance

When designing a national antimicrobial use surveillance system for implementation across hospitals in England, how well-suited would the system be if:

4	A large number of people (>4) were required to conduct surveillance in hospital	◯ 1 not at all suited<br>◯ 2<br>◯ 3<br>◯ 4<br>◯ 5 neither well-suited or not<br>◯ 6<br>◯ 7<br>◯ 8<br>◯ 9 extremely well-suited<br>◯ unsure<br>(For example, surveillance could be conducted by a small team of 1-4 professionals using digital data sets where they are available. Similarly a manual audit on a sample of the hospital population may require a small team. Conversely, hospital-wide manual audits often require a greater number of people to collect antimicrobial use data.)
5	Clinical training was required to collect the data in hospital	◯ 1 not at all suited<br>◯ 2<br>◯ 3<br>◯ 4<br>◯ 5 neither well-suited or not<br>◯ 6<br>◯ 7<br>◯ 8<br>◯ 9 extremely well-suited<br>◯ unsure<br>(For example, clinical training is required for a point prevalence survey which manually collects data on prescribing by interrogating patient notes. This is reflected in the key findings of the systematic review by the high proportion of physicians involved in this work. However, extensive clinical knowledge is not required to use digitally available datasets on prescribing linked to ICD codes to describe patterns of AMU. Is it suitable for an AMU surveillance strategy to expect hospitals to have clinically trained staff assigned to AMU surveillance work?)
6	Local data analytical skills were required to implement antimicrobial use monitoring in hospital, including professionals with the ability to use statistical packages such as R and STATA for analysis of big data sets on prescribing and patient care	◯ 1 not at all suited<br>◯ 2<br>◯ 3<br>◯ 4<br>◯ 5 neither well-suited or not<br>◯ 6<br>◯ 7<br>◯ 8<br>◯ 9 extremely well-suited<br>◯ unsure<br>(For example, this would be necessary if digital surveillance of AMU using e-prescribing systems were implemented. However, manual surveys on prescribing would not require as much expertise in handling big data sets.)

Outputs of surveillance

When designing a national antimicrobial use surveillance system for implementation across hospitals in England, how well-suited would the system be if:

7 The system to monitor antimicrobial use could support existing initiatives such as the CQUIN or national surveillance

- ◯ 1 not at all suited
- ◯ 2
- ◯ 3
- ◯ 4
- ◯ 5 neither well-suited or not
- ◯ 6
- ◯ 7
- ◯ 8
- ◯ 9 extremely well-suited
- ◯ unsure

8 There were an evidence base that implementing the system to monitor antimicrobial use in hospital leads to improved clinical outcomes

- ◯ 1 not at all suited
- ◯ 2
- ◯ 3
- ◯ 4
- ◯ 5 neither well-suited or not
- ◯ 6
- ◯ 7
- ◯ 8
- ◯ 9 extremely well-suited
- ◯ unsure

(For example, 54 (37.5%) of the studies captured in the systematic review used some form of audit and feedback on prescribing to improve AMU.)

9 The system to monitor antimicrobial use was integrated within existing hospital quality improvement initiatives such as reducing medication errors and improving sepsis outcomes

- ◯ 1 not at all suited
- ◯ 2
- ◯ 3
- ◯ 4
- ◯ 5 neither well-suited or not
- ◯ 6
- ◯ 7
- ◯ 8
- ◯ 9 extremely well-suited
- ◯ unsure

(For example, point prevalence surveys on antimicrobial use could also retrospectively monitor the management of sepsis, or the implementation of electronic prescribing systems to reduce medication errors could also be used for antimicrobial use surveillance.)

10 The antimicrobial use surveillance data could be used to compare antimicrobial use across specialties and hospitals

- ◯ 1 not at all suited
- ◯ 2
- ◯ 3
- ◯ 4
- ◯ 5 neither well-suited or not
- ◯ 6
- ◯ 7
- ◯ 8
- ◯ 9 extremely well-suited
- ◯ unsure

11	The antimicrobial use surveillance system monitored patient-level use over time, meaning that it is possible to conduct longitudinal studies	◯ 1 not at all suited<br>◯ 2<br>◯ 3<br>◯ 4<br>◯ 5 neither well-suited or not<br>◯ 6<br>◯ 7<br>◯ 8<br>◯ 9 extremely well-suited<br>◯ unsure

Page 5/9

Outputs of surveillance continued

When designing a national antimicrobial use surveillance system for implementation across hospitals in England, how well-suited would the system be if:

12 The measures collected were reported to high-level policy makers and they were used to inform decision-making

- ◯ 1 not at all suited
- ◯ 2
- ◯ 3
- ◯ 4
- ◯ 5 neither well-suited or not
- ◯ 6
- ◯ 7
- ◯ 8
- ◯ 9 extremely well-suited
- ◯ unsure

13 The measures collected were reported to Trust-level stakeholders who engaged with the surveillance system and used the measures to inform decision-making

- ◯ 1 not at all suited
- ◯ 2
- ◯ 3
- ◯ 4
- ◯ 5 neither well-suited or not
- ◯ 6
- ◯ 7
- ◯ 8
- ◯ 9 extremely well-suited
- ◯ unsure

14 The measures collected were reported to clinicians, who used them to inform prescribing decision-making

- ◯ 1 not at all suited
- ◯ 2
- ◯ 3
- ◯ 4
- ◯ 5 neither well-suited or not
- ◯ 6
- ◯ 7
- ◯ 8
- ◯ 9 extremely well-suited
- ◯ unsure

Page 6/9

Risk of implementing surveillance

When designing a national antimicrobial use surveillance system for implementation across hospitals in England, how well-suited would the system be if:

15 There was a lower risk of breach of confidentiality relating to patients, compared to other surveillance approaches

- ◯ 1 not at all suited
- ◯ 2
- ◯ 3
- ◯ 4
- ◯ 5 neither well-suited or not
- ◯ 6
- ◯ 7
- ◯ 8
- ◯ 9 extremely well-suited
- ◯ unsure

(For example, paper based surveillance systems with patient-level data pose a greater risk than secure, digitally available data sets)

16 There was a lower risk of misinterpreting the data than other surveillance approaches

- ◯ 1 not at all suited
- ◯ 2
- ◯ 3
- ◯ 4
- ◯ 5 neither well-suited or not
- ◯ 6
- ◯ 7
- ◯ 8
- ◯ 9 extremely well-suited
- ◯ unsure

(For example, without collecting information on patient case mix to describe patterns of prescribing, there is a risk of misinterpreting the data and inaccurately labelling clinicans as 'high prescribers' without considering their patient's needs. Similarly, when comparing hospital-level estimates to identify where there are higher than expected rates of prescribing, it would may be more meaningful to compare similar hospitals than to rank all hospitals in England against each other.)

17 There was a lower risk of unintended consequences of surveillance on prescriber behaviour

- ◯ 1 not at all suited
- ◯ 2
- ◯ 3
- ◯ 4
- ◯ 5 neither well-suited or not
- ◯ 6
- ◯ 7
- ◯ 8
- ◯ 9 extremely well-suited
- ◯ unsure

(For example, labelling clinicians as 'high prescribers' may lead to a shift in antimicrobial use (without optimising use) to use antimicrobials which are not being monitored and may not be concordant with guidelines. Conversely reporting measures at the hospital level, or not feeding the measures back to clinicians, may have a lower risk of these kinds of unintended consequences.)

National-level considerations for surveillance

When designing a national antimicrobial use surveillance system for implementation across hospitals in England, how well-suited would the system be if:

18 The system to monitor antimicrobial use could be implemented across hospitals with varying levels of resources including level of digital maturity

- ◯ 1 not at all suited
- ◯ 2
- ◯ 3
- ◯ 4
- ◯ 5 neither well-suited or not
- ◯ 6
- ◯ 7
- ◯ 8
- ◯ 9 extremely well-suited
- ◯ unsure

(For example, a range of paper and digital systems are in use across hospitals for patient care, including prescribing. This can act as a barrier to implementing single systems for AMU surveillance. One study referenced issues with linking data from different digital systems on prescribing and indication to microbiology for AMU surveillance.)

19 It required the diversion of funds away from other areas of antimicrobial stewardship

- ◯ 1 not at all suited
- ◯ 2
- ◯ 3
- ◯ 4
- ◯ 5 neither well-suited or not
- ◯ 6
- ◯ 7
- ◯ 8
- ◯ 9 extremely well-suited
- ◯ unsure

Page 8/9

Final questions

20 Please rank each approach to surveillance in order of the most well-suited to a national method for monitoring antimicrobial use (1) to least well-suited (3)

Manual Surveillance	◯ 1<br>◯ 2<br>◯ 3
Digital surveillance	◯ 1<br>◯ 2<br>◯ 3
Combination of manual and digital surveillance	◯ 1<br>◯ 2<br>◯ 3
Do you think any other factors are important to consider when developing a national antimicrobial use surveillance strategy?	__________________________________
Do you have anything further you would like to add?	__________________________________

Page 9/9
