# Supplementary material for: Enhancing antimicrobial surveillance in hospitals in England: a RAND-modified Delphi
Source: JAC Antimicrob Resist. 2022 Sep 12;4(5):dlac092. doi: 10.1093/jacamr/dlac092 (PMC9465639; doi:10.1093/jacamr/dlac092)
Supplement: dlac092_Supplementary_Data [file dlac092_supplementary_data.zip › Suppl2_evidence_summary_for_panel (4).pdf]

Antimicrobial use surveillance in hospitals

This information is based on data from a systematic review of studies which estimate antimicrobial use (AMU) in at least 100 hospital patients in high income countries. Data on surveillance approaches were extracted from 144 studies, including: sources and methods of data collection, professionals involved in surveillance and time taken. This primer outlines key findings from this review to inform the Delphi process. **Please use this information and your own professional experiences of AMU monitoring in English hospitals to answer the survey questions.**

Different approaches to measure antimicrobial use

There are 3 ways to obtain information on AMU in hospitals: manual, digital or a combination of both.

I. Manual data extraction and assessments of appropriateness of use have been used to contribute to national and international point and period prevalence surveys. Collating national survey data can lead to a delay in reports becoming available. Cohort studies have been conducted retrospectively or through daily chart review.

Using manual data extraction to sample a subset of the population may not be generalisable to the whole hospital population and may not provide information to target resources for stewardship unlike hospital-wide prevalence surveys.

A disadvantage of hospital-wide point prevalence surveys is that they are infrequent while regular surveys are labour intensive. Continuous surveillance and appropriateness assessments are preferable to prevalence surveys to better monitor trends over time and respond to inappropriate prescribing. Real-time assessments of appropriateness can then be used to make stewardship recommendations to inappropriate prescriptions.

II. Digital data extraction and assessments of appropriateness of use using AMU data collected by existing systems can be less labour intensive than manual audits. However not all hospitals will have digital systems for prescribing. The range of systems in use for different aspects of patient care across the hospital can also present a barrier to digitising surveillance. For example, one study cited that despite having access to ICD-9 codes and antibiotic exposure by linking pharmacy and billing systems, microbiology data could not be accessed. This can mean that a **combination of digital and manual data collection is required for surveillance (Table 2).**

Time and resources for antimicrobial use surveillance

I. Manual data extraction

Extracting information from patient notes and assessing appropriateness of AMU may require individuals with clinical expertise, i.e. **physicians**, to interpret information on indication linked to AMU. Large point prevalence surveys **require individuals to be trained** to standardise data collection

Time to extract data varies. The **European Surveillance of Antibiotic Consumption (ESAC) protocol** is estimated **10 mins per patient**. The **European Centre for Disease Control (ECDC) protocol** took **16 hrs of planning** in one hospital, followed by data extraction and a **further 16 hours for data cleaning and analysis**. A separate study estimated **45 mins per patient** for a research nurse/physician to extract data on prescribing and covariates.

II. Manual assessment of appropriateness

In order to standardise assessments of appropriateness, some studies apply previously published methods; one stated they used “clear cut” guidelines to avoid ambiguity or disagreement and others assessed concordance of therapy with available guidelines. Pre-defined criteria have been used to make assessments to contribute to national studies as well as local studies.

Time estimates to assess appropriateness vary. A number of studies estimate around 20 mins per patient and 5-10 minutes using electronic records. Other studies report 1 FTE pharmacist allocated to review all prescriptions at the site.

Different **professional backgrounds** may differ in inter-rater reliability and the benefit of a physician doing this work is an independent clinical assessment to determine infection, level of evidence of infection and therapy appropriateness.

III. Digital data extraction

Pharmacy dispensing system data is less labour intensive than manual data extraction, however this may not be patient-level data and lack covariates to adjust for patient case-mix

Reported limitations of electronic prescribing and medical administration system data include poor indication recording.

Algorithms can be used for data extraction and analysis which may reduce labour intensiveness and the time needed from clinical staff.

IV. Digital assessment of appropriateness

Algorithms can assess appropriateness but they are complex to develop and may need to be developed to be site specific using the data and guidelines available.

Professionals involved and data sources used for data collection and extraction

Table 1. Professionals involved in data collection by method of data extraction across the studies[a, b]
A certain level of clinical expertise is required to link prescription indication to AMU from patient notes. This is reflected in the high prevalence of physicians and pharmacists involved in this work.

Method of data extraction / Professional	Manual extraction, n = 109 (74%)	Manual + digital, n=8 (5%)	Digital extraction, n=21 (14%)	Not stated, n = 10 (7%)
Pharmacist	38 (26)	3 (2)	3 (2)	2 (1)
Physician	39 (26)	2 (1)	3 (2)	2 (1)
Nurse	13 (9)	0 (0)	1 (1)	0 (0)
Researcher	8 (6)	1 (1)	1 (1)	1 (1)
Antimicrobial management/stewardship teams or infection control teams (members not detailed by profession)	6 (4)	0 (0)	0 (0)	0 (0)
Microbiologist	4 (3)	0 (0)	0 (0)	0 (0)
Government body	1 (1)	0 (0)	0 (0)	0 (0)
Laboratory staff	0 (0)	0 (0)	1 (1)	0 (0)
Unknown hospital staff	15 (10)	2 (1)	4 (3)	2 (1)
Not stated	14 (9)	1 (1)	12 (8)	5 (3)

[a]Studies with more than one type of professional involved in antimicrobial use monitoring have been counted as many times.
[b]Studies with more than one method of data collection used across sites for antimicrobial use monitoring, have been included as many times.

Table 2. Method of data extraction and data sources used for the studies[c]
A combination of digital and paper data sources and methods of data extraction were used to estimate AMU. The majority of studies used manual data collection. Digital systems are necessary to digitally extract datasets on use, however some studies manually extracted data from digital systems. Facilitative data governance pathways and professionals skilled in data analysis must be in place to be able to harness the opportunity of digitally available datasets.

Method of Data Collection	Data Sources	Frequency, n (%)
Manual , n = 109 (74%)	Paper patient notes / prescriptions	30 (20)
	Electronic health records / prescriptions / pharmacy dispensing data / billing data / hospital census data/routine audit data/hospital data warehouse	18 (12)
	Combination of digital and paper	11 (7)
	Digital and another not specified or unclear data source	4 (3)
	Paper and another not specified or unclear data source	1 (1)
	Not specified or unclear data source	45 (30)
Digital, n = 21 (14%)	Electronic health records / prescriptions / pharmacy dispensing data / billing data / hospital census data/routine audit data/hospital data warehouse	21 (14)
Combined manual and digital, n = 8 (5%)	Electronic health records / prescriptions / pharmacy dispensing data / billing data / hospital census data/routine audit data/hospital data warehouse	3 (2)
	Combination of digital and paper	2 (1)
	Digital and another not specified or unclear	3 (2)
Not specified or unclear, n = 10 (7%)	Electronic health records / prescriptions / pharmacy dispensing data / billing data / hospital census data/routine audit data/hospital data warehouse	2 (1)
	Digital and another not specified or unclear	1 (1)
	Not specified or unclear	7 (5)

[c]Studies with more than one combination of method of data collection and data sources used across sites for antimicrobial use monitoring, have been included as many times.
