# Supplementary material for: Enhancing antimicrobial surveillance in hospitals in England: a RAND-modified Delphi
Source: JAC Antimicrob Resist. 2022 Sep 12;4(5):dlac092. doi: 10.1093/jacamr/dlac092 (PMC9465639; doi:10.1093/jacamr/dlac092)
Supplement: dlac092_Supplementary_Data [file dlac092_supplementary_data.zip › Suppl3_pannelist_Round1_reminder (4).pdf]

Delphi process on antimicrobial use surveillance strategies in hospitals in England:

Responses to Questionnaire 1

This document is to inform you of how your responses to the first questionnaire of this process, compares against those from the rest of the group (median). This is to inform and aid the group discussion about these questions. *Please ensure you have this with you for the group conference call*.

Description of the expert group

Description	Frequency (n = 24)
**Profession**	
Microbiologist	8
Pharmacist	14
Physician	1
Public Health	1
**Average years since qualification**	
	22 years
**Members of stewardship groups**	
Local	12
National	12
**Region of England**	
National	2
South West	2
North East	7
Midlands	3
London	9
East of England	1
**Hospital type (if applicable)**	
District General	7
Specialty hospital	1
University hospital	14
**Hospital use of electronic prescribing or electronic health records for patient care**	
Yes	18
No	4
**Those working for an organisation affiliated with government**	
	2

Reminder of the Likert scale for responses

1	2	3	4	5	6	7	8	9

not at all suited — neither well-suited or not — extremely well suited

Participants could also respond as ‘*unsure’*

Resources required for surveillance

For each question, the response which you gave is marked with a *.

When designing a national antimicrobial use surveillance system for implementation across hospitals in England, how well-suited would the system be if:

1. Longer than a day was required to establish the system in hospital to monitor antimicrobial use

Group median response: 5 | range = 3 – 9 | 2 were unsure

**Response**	**1**	**2**	**3**	**4**	**5**	**6**	**7**	**8**	**9**	**unsure**
**Frequency**	0	0	2	3	9	1	3	3	1	2

2. Longer than a day was required to conduct the antimicrobial use surveillance once the monitoring system is in place

Group median response: 3 | range = 2 – 9 | 0 were unsure

**Response**	**1**	**2**	**3**	**4**	**5**	**6**	**7**	**8**	**9**	**unsure**
**Frequency**	0	7	8	2	2	3	1	0	1	0

3. High costs were involved in the set up and maintenance of the system to monitor antimicrobial use in hospital

Group median response: 3 | range = 1 – 8 | 0 were unsure

**Response**	**1**	**2**	**3**	**4**	**5**	**6**	**7**	**8**	**9**	**unsure**
**Frequency**	3	8	2	7	2	1	0	1	0	0

Implementing surveillance

When designing a national antimicrobial use surveillance system for implementation across hospitals in England, how well-suited would the system be if:

4. A large number of people (>4) were required to conduct surveillance in hospital

Group median response: 3 | range = 1 – 8 | 0 were unsure

**Response**	**1**	**2**	**3**	**4**	**5**	**6**	**7**	**8**	**9**	**unsure**
**Frequency**	4	4	5	5	4	1	0	1	0	0

5. Clinical training was required to collect the data in hospital

Group median response: 5 | range = 1 – 8 | 0 were unsure

**Response**	**1**	**2**	**3**	**4**	**5**	**6**	**7**	**8**	**9**	**unsure**
**Frequency**	2	2	3	2	5	7	2	1	0	0

6. Local data analytical skills were required to implement antimicrobial use monitoring in hospital, including professionals with the ability to use statistical packages such as R and STATA for analysis of big data sets on prescribing and patient care

Group median response: 3 | range = 1 – 7 | 0 were unsure

Response	1	2	3	4	5	6	7	8	9	unsure
Frequency	5	3	5	5	3	2	1	0	0	0

Outputs of surveillance

When designing a national antimicrobial use surveillance system for implementation across hospitals in England, how well-suited would the system be if:

7. The system to monitor antimicrobial use could support existing initiatives such as the CQUIN or national surveillance

Group median response: 9 | range = 1 – 9 | 0 were unsure

Response	1	2	3	4	5	6	7	8	9	unsure
Frequency	1	0	0	0	0	1	3	5	14	0

8. There were an evidence base that implementing the system to monitor antimicrobial use in hospital leads to improved clinical outcomes

Group median response: 9 | range = 6 – 9 | 0 were unsure

Response	1	2	3	4	5	6	7	8	9	unsure
Frequency	0	0	0	0	0	1	3	5	15	0

9. The system to monitor antimicrobial use was integrated within existing hospital quality improvement initiatives such as reducing medication errors and improving sepsis outcomes

Group median response: 9 | range = 5 – 9 | 1 was unsure

Response	1	2	3	4	5	6	7	8	9	unsure
Frequency	0	0	0	0	2	1	2	5	13	1

10. The antimicrobial use surveillance data could be used to compare antimicrobial use across specialties and hospitals

Group median response: 8 | range = 1 – 9 | 0 were unsure

Response	1	2	3	4	5	6	7	8	9	unsure
Frequency	1	0	0	0	1	2	6	4	10	0

11. The antimicrobial use surveillance system monitored patient-level use over time, meaning that it is possible to conduct longitudinal studies

Group median response: 8 | range = 1 – 9 | 0 were unsure

Response	1	2	3	4	5	6	7	8	9	unsure
Frequency	1	0	0	0	0	3	6	5	9	0

Outputs of surveillance continued

When designing a national antimicrobial use surveillance system for implementation across hospitals in England, how well-suited would the system be if:

12. The measures collected were reported to high-level policy makers and they were used to inform decision-making

Group median response: 8 | range = 3 - 9 | 0 were unsure

Response	1	2	3	4	5	6	7	8	9	unsure
Frequency	0	0	1	2	1	1	4	8	7	0

13. The measures collected were reported to Trust-level stakeholders who engaged with the surveillance system and used the measures to inform decision-making

Group median response: 8 | range = 4 - 9 | 0 were unsure

Response	1	2	3	4	5	6	7	8	9	unsure
Frequency	0	0	0	1	0	1	5	7	10	0

14. The measures collected were reported to clinicians, who used them to inform prescribing decision-making

Group median response: 8 | range = 4 - 9 | 0 were unsure

Response	1	2	3	4	5	6	7	8	9	unsure
Frequency	0	0	0	1	0	2	3	7	11	0

Risk of implementing surveillance

When designing a national antimicrobial use surveillance system for implementation across hospitals in England, how well-suited would the system be if:

15. There was a lower risk of breach of confidentiality relating to patients, compared to other surveillance approaches

Group median response: 8 | range = 5 - 9 | 0 were unsure

Response	1	2	3	4	5	6	7	8	9	unsure
Frequency	0	0	0	0	2	2	4	5	11	0

16. There was a lower risk of misinterpreting the data than other surveillance approaches

Group median response: 8.5 | range = 7 - 9 | 0 were unsure

Response	1	2	3	4	5	6	7	8	9	unsure
Frequency	0	0	0	0	0	0	5	7	12	0

17. There were a lower risk of unintended consequences of surveillance on prescriber behaviour

Group median response: 8 | range = 2 - 9 | 2 were unsure

Response	1	2	3	4	5	6	7	8	9	unsure
Frequency	0	1	0	0	2	1	4	8	6	2

18. The system to monitor antimicrobial use could be implemented across hospitals with varying levels of resources including level of digital maturity

Group median response: 8.5 | range = 2 - 9 | 0 were unsure

Response	1	2	3	4	5	6	7	8	9	unsure
Frequency	0	1	0	1	0	1	4	5	12	0

19. It required the diversion of funds away from other areas of antimicrobial stewardship

Group median response: 4.5 | range = 2 - 9 | 0 were unsure

Response:	1	2	3	4	5	6	7	8	9	unsure
Frequency	0	3	1	8	7	4	0	0	1	0

Additional suggestions put forward by experts

Should the following also be discussed in this process to consider the suitability of existing approaches for application in a national surveillance strategy?

1. [For manual strategies] Time of year for the survey
2. [For manual strategies] peer review of data entry processes to ensure integrity
3. Several responses queried how to account for differences in case mix, for example:
    a. The provision of specialty services should be accounted for when estimating rates of prescribing
    b. Trust prescribing guidelines also influence the data and this needs to be accounted for
4. Robust mechanisms for feedback to clinicians “which address the issues of hierarchy within the NHS”
    a. Furthermore – ensuring that what the system measures is linked to the improvements we are aiming for, as well as ensuring that everyone is engaged with stewardship through appraisal
5. To achieve clinician-level monitoring, the system should clearly lay out how to attribute prescriptions as several teams will be involved in managing one prescription throughout the patient’s stay in hospital
6. The system must be versatile to enable hospitals to measure what is important locally, so as not to get ‘stuck’ reporting what experts agree is important nationally
    a. Ensure that the system provides data of relevance to the organisations taking part and the clinical teams who will see the data
    b. Data is appropriately benchmarked and benchmarks are ‘achievable’
7. The system must be properly piloted across several settings to ensure that it is suitable
8. The system should measure both quantitative consumption and quality of use such as prescription review
9. Should not be seen as having potential to unfairly criticise those who appear not to be performing well

10. [For digital systems, they must] demonstrate compatibility with all e-prescribing systems currently available in the UK
